# The genome of the rayed Mediterranean limpet *Patella caerulea* (Linnaeus, 1758)

**DOI:** 10.1093/gbe/evae070

**Published:** 2024-03-28

**Authors:** Gwyneth Halstead-Nussloch, Silvia Giorgia Signorini, Marco Giulio, Fabio Crocetta, Marco Munari, Camilla Della Torre, Alexandra Anh-Thu Weber

**Affiliations:** Department of Environmental Systems Science, ETH Zürich, Zürich, Switzerland; Department of Aquatic Ecology, Swiss Federal Institute of Aquatic Science and Technology (Eawag), Dübendorf, Switzerland; Department of Biosciences, University of Milan, Milan, Italy; Department of Integrative Marine Ecology, Stazione Zoologica Anton Dohrn, Naples, Italy; Department of Aquatic Ecology, Swiss Federal Institute of Aquatic Science and Technology (Eawag), Dübendorf, Switzerland; Department of Integrative Marine Ecology, Stazione Zoologica Anton Dohrn, Naples, Italy; National Biodiversity Future Center (NBFC), Palermo, Italy; Department of Integrative Marine Ecology, Stazione Zoologica Anton Dohrn, Naples, Italy; Department of Biology, Stazione Idrobiologica ‘Umberto d’Ancona’, University of Padova, Chioggia, Italy; Department of Biosciences, University of Milan, Milan, Italy; Department of Integrative Marine Ecology, Stazione Zoologica Anton Dohrn, Naples, Italy; Department of Aquatic Ecology, Swiss Federal Institute of Aquatic Science and Technology (Eawag), Dübendorf, Switzerland

**Keywords:** assembly, annotation, RNAseq, PacBio HiFi, ERGA, mollusc

## Abstract

*Patella caerulea* (Linnaeus, 1758) is a mollusc limpet species of the class Gastropoda. Endemic to the Mediterranean Sea, it is considered a keystone species due to its primary role in structuring and regulating the ecological balance of tidal and subtidal habitats. It is currently being used as a bioindicator to assess the environmental quality of coastal marine waters and as a model species to understand adaptation to ocean acidification. Here, we provide a high-quality reference genome assembly and annotation for *P. caerulea*. We generated ∼30 Gb of Pacific Biosciences high-fidelity data from a single individual and provide a final 749.8 Mb assembly containing 62 contigs, including the mitochondrial genome (14,938 bp). With an N50 of 48.8 Mb and 98% of the assembly contained in the 18 largest contigs, this assembly is near chromosome-scale. Benchmarking Universal Single-Copy Orthologs scores were high (Mollusca, 87.8% complete; Metazoa, 97.2% complete) and similar to metrics observed for other chromosome-level *Patella* genomes, highlighting a possible bias in the Mollusca database for Patellids. We generated transcriptomic Illumina data from a second individual collected at the same locality and used it together with protein evidence to annotate the genome. A total of 23,938 protein-coding gene models were found. By comparing this annotation with other published *Patella* annotations, we found that the distribution and median values of exon and gene lengths was comparable with other *Patella* species despite different annotation approaches. The present high-quality *P. caerulea* reference genome, available on GenBank (BioProject: PRJNA1045377; assembly: GCA_036850965.1), is an important resource for future ecological and evolutionary studies.

**Significance**

Reference genomes are essential resources for biodiversity conservation and management. *Patella caerulea* (*Linnaeus*, 1758) is a gastropod species occurring in the Mediterranean Sea that is currently used as a model to understand the impact of pollution and ocean acidification on marine biodiversity. Here, we present a high-quality reference genome of *P. caerulea* that almost reaches chromosome-level contiguity. We further provide a high-quality genome annotation supported by transcriptomic evidence. This reference genome will be of interest for researchers working on the ecology and evolution of marine biodiversity. All data are available on the public database National Center for Biotechnology Information (NCBI) for future use by researchers.

## Introduction

Patellid limpets (family Patellidae Rafinesque, 1815) are gastropod molluscs, containing ∼400 species distributed worldwide. Patellid species are divided into four different genera (Ridgway et al. 1998; Koufopanou et al. 1999). Species of the genus *Patella* mostly occur in the northeastern Atlantic and the Mediterranean Sea, and this genus currently includes 16 valid species. Some of these species have a very limited distribution and are endemic to isolated islands and archipelagos (Titselaar 2019), while others have a wider distribution, being widespread in the Atlantic Ocean and/or the Mediterranean Sea (Gofas et al. 2011; Alf et al. 2020). However, the correct taxonomy of some taxa is still under investigation, as they may account for yet-unsolved species complexes (Sá-Pinto et al. 2010).

**Fig. 1.**
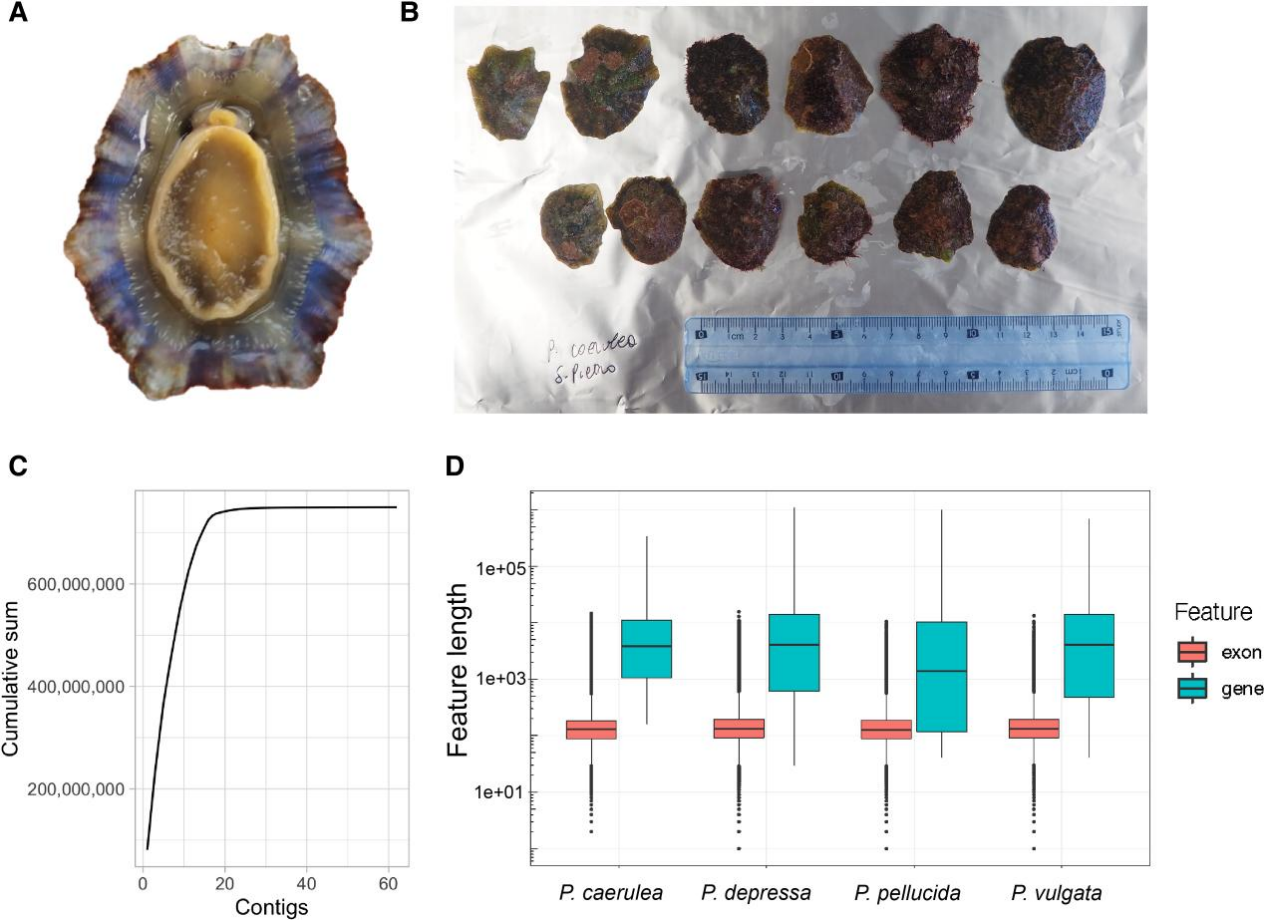
*P. caerulea* reference specimens and reference genome. A) Ventral view of a typical specimen of *P. caerulea*. B) Dorsal view of *P. caerulea* specimens collected in San Pietro, Italy. This is the same locality where the *P. caerulea* individuals used for genome and transcriptome sequencing were collected. C) Cumulative sum of contig length (bp), ordered by contig length. D) Annotation feature comparison with other *Patella* annotations available on NCBI. Distribution and median values for gene and exon length are similar between species.

The rayed Mediterranean limpet *Patella caerulea* (Linnaeus, 1758) is endemic to the Mediterranean Sea and widespread from the western to the eastern shores of the basin (Grossu 1993; Gofas et al. 2011; Crocetta et al. 2020). Shell morphological traits of this species are highly variable, with the presence of different morphs depending on ontogenic or ecophenotypic variables (Cossignani and Ardovini 2011; Gofas et al. 2011). This is reflected in a long list of synonyms and even varieties described by past authors, now all synonymized under the valid nominal taxon (MolluscaBase eds. 2023). Notwithstanding such limitations, *P. caerulea* is generally characterized by an external greyish–brownish shell, with darker radiating bands and thin radial costae, and by the presence of a bluish iridescent layer internally, often well evident and from which the species is named (Fig. 1A and B).

The species is protandrous hermaphrodite and is considered a predominantly winter breeder (Ferranti et al. 2018). *P. caerulea* lives attached to rocks, from the lower intertidal to the subtidal zone, usually up to 5 m depth. It is considered a keystone species due to its primary role in structuring and regulating the ecological balance of intertidal communities, both directly through grazing and acting as prey for higher trophic-level consumers and indirectly by enhancing or inhibiting the settlement of other organisms (Menge 2000; Reguera et al. 2018; Vafidis et al. 2020; Aydin et al. 2021). Similar to other limpets, the species is threatened by different environmental and anthropogenic disturbances, which include ocean acidification, rising temperature, and pollution (Vafidis et al. 2020).

Given the wide distribution and abundance, sedentary lifestyle, and key ecological role, *P. caerulea* has been used as bioindicator in studies aimed at assessing the environmental quality of coastal marine waters, through the measurement of bioaccumulation in tissues and the adverse biological effects of different classes of xenobiotics (Aydın-Önen and Öztürk 2017; Reguera et al. 2018; Zaidi et al. 2022). In this context, a study investigated the potential influence of coastal urbanization on the genetic variation of *P. caerulea* (Fauvelot et al. 2009). This study highlighted the fact that coastal urbanization may act as a biotic homogenization, reducing genetic diversity of local species, with consequences on productivity, growth, stability, and interactions at both community and ecosystem levels. Finally, *P. caerulea* is currently used as a model to understand adaptation to ocean acidification in gastropods, as natural populations adapted to reduced pH conditions (∼7.4) have been discovered in the CO_2_ vent systems of Ischia Island, Italy, together with *Patella ulyssiponensis* and *Patella rustica* (Aliende et al. 2023). Specifically, individuals of these three species occurring in reduced pH conditions have thinner shells and larger sizes than individuals from ambient pH conditions. However, the specific molecular mechanisms underlying this phenotypic variability are unknown. In particular, the respective influence of phenotypic plasticity versus genetic adaptation has not been examined yet and is currently under investigation.

Despite its important ecological and evolutionary roles and its use as a bioindicator, there is to date no reference genome for *P. caerulea*. There are currently four reference genome projects of *Patella* species, three that are published and assembled to the chromosome level (*Patella depressa*, *Patella vulgata*, *Patella pellucida*) (Lawniczak et al. 2022; Hawkins et al. 2023) and one (*P. ulyssiponensis*), led by the Darwin Tree of Life initiative, for which the chromosome-scale genome assembly has been recently released (ENA project PRJEB63446), but the genome note has to date not been published (Table 1). These *Patella* species are diploid, have between eight and nine pairs of chromosomes (Petraccioli et al. 2010), and have assembly sizes varying between 683 and 712 Mb. *P. caerulea* also has nine pairs of chromosomes (Petraccioli et al. 2010), but there are no genome size estimates for this species.

**Table 1:** Metrics of the *P. caerulea* PatCaer1 genome assembly and comparison with publicly available Patella genome projects

Species	Reference genome	GenBank/ENA accession number	Assembly size	# nuclear linkage groups	# scaffolds/contigs	Scaffold/contig N50	BUSCO score Mollusca database (odb_10, n:5295)	BUSCO score Metazoa database (odb_10, n:954)	Reference
*P. caerulea*	PatCaer1	GCA_036850965.1	749.8 Mb	NA	62	48.8 Mb	C: 87.8% (S: 83.8%, D: 4.0%), F: 5.2%, M: 7.0%	C: 97.2% (S: 93.5%, D: 3.7%), F: 1.8%, M: 1.0%	This study
	

*P. depressa*	xgPatDepr1.1	GCA_948474765.1	683.7 Mb	9	35	77.5 Mb	C: 88.2% (S: 87.3%; D: 0.9%), F: 5.3%; M: 6.5%	C: 98.0% (S: 97.4%; D: 0.6%) F: 1.4%; M: 0.6%	Hawkins et al. 2024
	

*P. vulgata*	xgPatVulg1.2	GCA_932274485.2	695.4 Mb	9	22	87.4 Mb	C: 88.3% (S: 87.3%, D: 0.9%), F: 5.1%, M: 6.6%	C: 97.6% (S: 96.9%, D: 0.7%), F: 1.6%, M: 0.8%	Hawkins et al. 2023
	
	
*P. pellucida*	xgPatPell1.1	GCA_917208275.1	712 Mb	9	62	87.2 Mb	C: 87.6% (S: 86.7%, D: 0.8%), F: 5.2%, M: 7.3%	C: 97.5% (S: 96.9%, D: 0.6%), F: 1.6%, 0.9%	Lawniczak et al. 2022
	
	
*P. ulyssiponensis* ^a^	xgPatUlys2.1	GCA_963678685.1	693.6 Mb	8	27	86.7 Mb	C: 88.2% (S: 86.9%, D: 1.3%), F: 5.0%, M: 6.8%	NA	BioProject PRJEB70253
	


^a^BUSCO scores may change as the *P. ulyssiponensis* genome note is unpublished yet (status 2024 March 12).

Having high-quality reference genomes is essential for the broad field of biodiversity genomics (Formenti et al. 2022; Theissinger et al. 2023). As a result, numerous initiatives and consortia are now aiming at generating high-quality reference genomes for all described eukaryotic species on Earth (e.g. the Earth Biogenome Project—EBP) (Lewin et al. 2018, 2022). The European Reference Genome Altas (ERGA) initiative is the European node of the EBP, which aims at sequencing and assembling the genomes of all eukaryotic species in Europe (Mazzoni et al. 2023; Mc Cartney et al. 2024). Within animals, while sequencing of vertebrates is advancing well (Hotaling et al. 2021; Rhie et al. 2021), genome projects on invertebrates, especially arthropods and molluscs, are comparatively lagging behind (Hotaling et al. 2021). Here, we provide a high-quality reference genome of *P. caerulea* which will help future ecological and evolutionary studies of the rayed Mediterranean limpet.

## Results and Discussion

### Genome Assembly

We used a single *P. caerulea* specimen to generate about 30 Gb of high-fidelity (HiFi) Pacific Biosciences (PacBio) data from a single SMRT cell. Genome size was estimated to be ∼623 Mb and heterozygosity of 2.06% using k-mer-based methods (supplementary fig. S1A, Supplementary Material online). Correctness (QV) was estimated to be 68.5 and k-mer completeness was 77.53% (supplementary fig. S1B, Supplementary Material online), similar to haploid assemblies of other heterozygous species (Qi et al. 2022). The final assembly PatCaer1 was 749.8 Mb in length and contained 62 contigs including the mitochondrial genome (Table 1, supplementary fig. S1C, Supplementary Material online). Assuming that the genome size is closer to the assembly size than the genome scope estimate, this results in an approximate sequencing coverage of 40×. A total of 98.4% of the assembly was contained in the 18 largest contigs (Fig. 1C), indicating an assembly close to chromosome level from HiFi reads alone, given that *P. caerulea* has nine pairs of chromosomes. Benchmarking Universal Single-Copy Orthologs (BUSCO) completeness was 87.8% using the mollusca_odb10 and 97.2% with metazoa_odb10 reference set, a pattern also observed for at least three other *Patella* species (Table 1), further indicating potential clade-specific biases in the mollusca reference set. This may indicate lineage-specific gene losses, which could also be the case for other species of the Patellidae family in future reference genome projects.

These metrics show that this very contiguous assembly provides a high-quality genomic resource for *P. caerulea* generated with data from a single sequencing technology (PacBio). A 14,938 bp contig corresponding to the mitochondrial genome was identified within the primary assembly by Tiara classification and confirmed using BLAST and presence of mitochondrial genes using MITOS2 (Donath et al. 2019). Another contig identified as mitochondrial by Tiara had subsequent BLAST hits with other *Patella* nuclear (i.e. ribosomal genes) but not mitochondrial genome sequences. We therefore kept this contig in the nuclear assembly.

### Genome Annotation

Gene models were generated using both RNA and protein evidence, which resulted in 23,938 protein-coding gene models (supplementary table S1, Supplementary Material online). This number is higher than the number of protein-coding genes identified in *P. vulgata*, where the most similar annotation pipeline was implemented (i.e. the use of BRAKER2) (Hawkins et al. 2023). BUSCO completeness was 90.7% using the mollusca_odb10 database and 96.0% with metazoa_odb10, similar or even higher than the completeness assessed for the genome assembly, which suggests high-quality annotation (Manni et al. 2021b). The distribution and median values of exon and gene lengths was comparable with other *Patella* species despite different annotation approaches, indicating congruence in structural feature identification within the *Patella* assemblies (Fig. 1D). It should be noted that annotations for the other *Patella* species include further classifications beyond protein-coding sequences that were not identified in this version of the *P. caerulea* annotation, increasing the number of gene and transcript features in those annotations (supplementary table S1, Supplementary Material online). Finally, we found 16,646 reciprocal best BLAST hits between *P. caerulea* and *P. depressa* amino acid sequences.

We note that while genome assembly metrics are easily comparable between projects and data sets, it is more difficult to compare genome annotations, even between species from the same genus. While there are initiatives to standardize annotations (e.g. ERGA recommendations, https://www.erga-biodiversity.eu/structural-annotation), it is currently difficult to directly compare annotations between species, especially nonmodel organisms. Indeed, annotation pipelines utilize different methods and availability (or lack thereof) of external evidence (e.g. RNAseq), which quickly generates discrepancies between each annotation project. Finally, we acknowledge that Hi-C data would add valuable information to further scaffold to the chromosome level and phase the present assembly. It would as well be useful for manual curation and correction of potential misassemblies. However, the present primary assembly is already highly contiguous and well-annotated, and we believe it will be a valuable resource for the community of researchers working on the ecology and evolution of *P. caerulea*.

## Materials and Methods

### Sample Collection, DNA and RNA Extractions, and Sequencing

Ten *P. caerulea* individuals were collected by snorkeling at 2 m depth on 2023 June 9 in Punta San Pietro, Ischia Island, Italy (40.74702561880045, 13.943306501480043) with the permission of the Marine Protected Area “Regno di Nettuno.” High-molecular weight DNA extraction from a single individual (National Center for Biotechnology Information [NCBI] Biosample SAMN38441487, PatCaer1) was performed using the Qiagen MagAttract HMW kit following manufacturer's instructions. The individual was not sexed. A single PacBio standard library (15 to 20 kb insert size) was constructed and sequenced on one SMRT cell 8M on a PacBio Sequel II instrument at the Functional Genomics Center Zürich (FGCZ), Switzerland. A second specimen (NCBI Biosample SAMN38441544, PatCaer2) was used for RNA extractions. Individual RNA extractions from head, foot/mantle, and visceral body were performed using the E.Z.N.A. HP Total RNA Isolation Kit (Omega Bio-tek). Three RNA extractions passing QC (RIN > 9) were pooled in equimolar concentrations and a single transcriptomic library was constructed using Truseq mRNA kit (Illumina) and sequenced on a Illumina NovaSeq 6000 sequencer (200 M reads, 150 bp paired-end) at the FGCZ.

### Genome Size Estimate and Genome Assembly

A total of 1,984,661 PacBio HiFi reads containing a total of 29,667,167,600 bases and a mean length of 14,948 bp were used for the assembly. Meryl v1.3 (Rhie et al. 2020) was used to generate a k-mer database. K-mer frequency was analyzed and genome size estimated by GenomeScope2 (Ranallo-Benavidez et al. 2020). A primary assembly was produced using hifiasm (Cheng et al. 2022) v0.19.6-r595 with purging parameter set to -s 0.35. Contigs were evaluated by (i) measuring GC content using asmstats (https://github.com/marcelauliano/Teaching/blob/main/asmstats) and seqtk v1.3-r117-dirty (https://github.com/lh3/seqtk), (ii) coverage of HiFi reads mapped back to the assembly using minimap2 (Li, 2018), and (iii) sequence classification by Tiara (Karlicki et al. 2021). Contigs not classified as eukaryotic or mitochondrial, with coverage below 5× or above 100×, were removed from the primary assembly. Assembly metrics were further evaluated using BUSCO v5.2.2 with both the mollusca and metazoa odb10 databases (Manni et al. 2021a). QV, k-mer completeness, and k-mer copy number plots were estimated and generated using merqury v1.3 (Rhie et al. 2020).

### Annotation

Prior to annotation, repeat libraries including LTR sequences were generated from the contigs using RepeatModeler2 (Flynn et al. 2020) and combined with mollusca-specific repeat family sequences from Dfam (Storer et al. 2021) prior to running RepeatMasker (Smit et al. 2013). The softmasked assembly was then used as input for the BRAKER pipelines (Hoff et al. 2016, 2019; Bruna et al. 2021) in order to generate gene models. Briefly, 200 M paired-end RNAseq reads were aligned to the masked assembly using HISAT2 (Kim et al. 2019) and the bam file was used as input for the BRAKER1 pipeline (Hoff et al. 2016). Metazoa-specific protein sequences partitioned from OrthoDB v11 (Kuznetsov et al. 2023) were used as input for the BRAKER2 pipeline (Bruna et al. 2021). The gene models in the braker.gtf output from both pipelines were combined using default evidence weights and the --ignore_tx_phase option and renamed using TSEBRA (Gabriel et al. 2021).

We followed some recommendations of the ERGA initiative to assess the quality of our annotation (https://www.erga-biodiversity.eu/structural-annotation). Specifically, we used BUSCO v5.2.2 with both the mollusca and metazoa odb10 databases (Manni et al. 2021a) to evaluate the completeness of our annotation, using the longest isoform for analysis. We also evaluated our annotation by comparing the distribution and median lengths of features classified as “exon” and “gene” among the species *P. caerulea*, *P. depressa*, *P. pellucida*, and *P. vulgata* for which annotation data is available on NCBI. Finally, we ran a reciprocal best BLAST hit analysis between the *P. caerulea* and *P. depressa* amino acid sequences to infer how many orthologous genes are present between both species.

## Supplementary Material

evae070_Supplementary_Data

## Data Availability

The genomic and transcriptomic raw data are available on NCBI Sequence Read Archive (SRA) BioProject ID PRJNA1045377 (https://www.ncbi.nlm.nih.gov/sra): Biosample IDs SAMN38441487 (for PacBio HiFi) and SAMN38441544 (for RNAseq); PacBio data, SRX22853381; and RNAseq data, SRX22853382. The PatCaer1 genome assembly and annotation (*P. caerulea* AATW-2023a) have been deposited on NCBI (https://www.ncbi.nlm.nih.gov/genome/) and are available under the accession number JAZGQO000000000 and assembly number GCA_036850965.1. The version described in this paper is version JAZGQO010000000. The commands used are available on the GitHub repository (https://github.com/GwynHN/PatCaer_asm_anno).
