# Supplementary material for: The genome of the rayed Mediterranean limpet Patella caerulea (Linnaeus, 1758)
Source: Genome Biol Evol. 2024 Mar 28;16(4):evae070. doi: 10.1093/gbe/evae070 (PMC11003540; doi:10.1093/gbe/evae070)
Supplement: evae070_Supplementary_Data [file evae070_supplementary_data.zip › Supplemental figure legend.docx]

Supplemental figure legend

**Figure S1. *Patella caerulea* PatCaer1 genome assembly and annotation metrics.** **A)** GenomeScope2 k-mer profile with estimates of genome size and heterozygosity. **B)** Merqury k-mer plot. Assembly k-mer copy number spectrum shows low level of duplications within the assembly and that most kmers are incorporated into the haploid assembly. Read-only k-mers in the single-copy peak are from alternate haplotypes from this heterozygous species. **C)** Contigs were evaluated by mapping HiFi reads back on the assembly and calculating mean coverage for each contig (y-axis: HiFi coverage). Contig average GC content (x-axis), Tiara sequence classification (circle color) and length (circle size) is also displayed.
