# Supplementary material for: The genome of the rayed Mediterranean limpet Patella caerulea (Linnaeus, 1758)
Source: Genome Biol Evol. 2024 Mar 28;16(4):evae070. doi: 10.1093/gbe/evae070 (PMC11003540; doi:10.1093/gbe/evae070)
Supplement: evae070_Supplementary_Data [file evae070_supplementary_data.zip › Table_S1_Halstead-Nussloch_et-al.docx]

**Table S1.** Annotation metrics of *P. caerulea* and comparison with published *Patella* genome annotations. See Table 1 for references.

| **Statistics** | ***P. caerulea*** | ***P. depressa*** | ***P. pellucida*** | ***P. vulgata*** |
| --- | --- | --- | --- | --- |
| Number of protein-coding genes | 23,938 | 20,502 | 18,161 | 19,378 |
| Number of transcripts | 29,627 | 40,054 | 53,889 | 47,283 |
| Mean gene length | 9,284 | 13,421 | 10,190 | 12,757 |
| Median gene length | 3,870 | 4,098 | 1,393 | 4,088 |
| Mean exon length | 208 | 211 | 201 | 214 |
| Median exon length | 129 | 133 | 127 | 132 |
| Mean exons per transcript | 7.09 | 6.25 | 5.55 | 6.54 |
| Median exons per transcript | 4 | 3 | 2 | 3 |
| BUSCO score Mollusca (odb10) | C:90.7%  [S:86.9%,D:3.8%],  F:0.8%,M:8.5%,n:5295 | NA | NA | NA |
| BUSCO score Metazoa (odb10) | C:96.0%  [S:92.2%,D:3.8%],  F:1.2%,M:2.8%,n:954 | NA | NA | NA |
