# Supplementary figures and images for: The genome of the rayed Mediterranean limpet Patella caerulea (Linnaeus, 1758)
Source: Genome Biol Evol. 2024 Mar 28;16(4):evae070. doi: 10.1093/gbe/evae070 (PMC11003540; doi:10.1093/gbe/evae070)

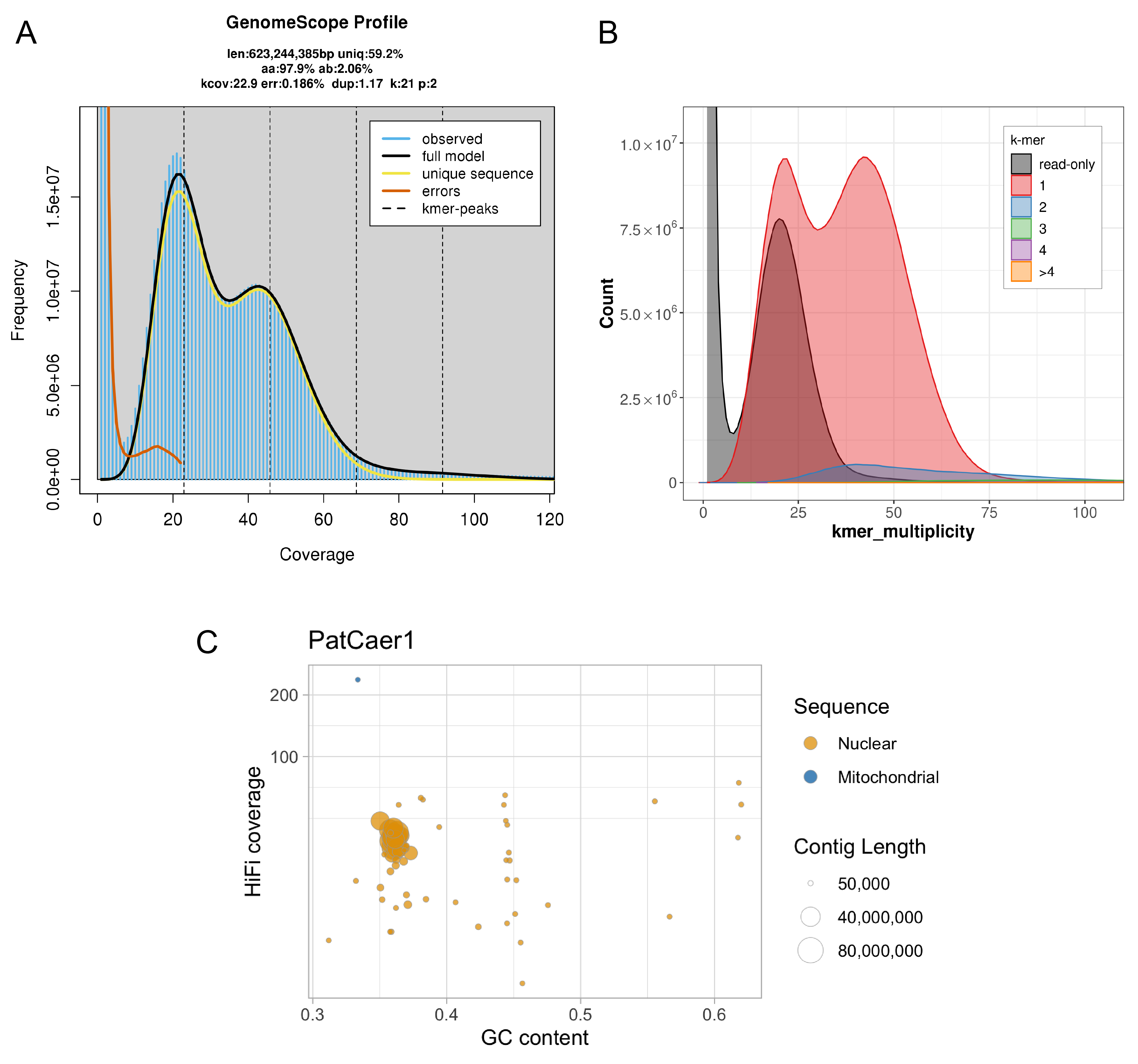

Supplement: evae070_Supplementary_Data [file evae070_supplementary_data.zip › Figure_S1_Halstead-Nussloch_et_al.png]
